# circATAD2 mitigates CD8^+^ T cells antitumor immune surveillance in breast cancer via IGF2BP3/m^6^A/PD-L1 manner

**DOI:** 10.1007/s00262-024-03705-6

**Published:** 2024-05-15

**Authors:** Zhiling Zhang, Wenjie Huo, Jie Li

**Affiliations:** Department of Breast Surgery, Shanxi Provincial People’s Hospital, Taiyuan, 030012 China

**Keywords:** Breast cancer, Immune surveillance, circATAD2, N^6^-methyladenosine, PD-L1

## Abstract

Immune surveillance and chemotherapy sensitivity play critical functions in the tumorigenesis of breast cancer (BC). Emerging findings have indicated that circular RNA (circRNA) and N^6^-methyladenosine (m^6^A) both participate in the BC tumorigenesis. Here, present study aimed to investigate the roles of m^6^A-modified circATAD2 on BC and explore better understanding for BC precision therapeutic. Results reported that m^6^A-modifid circRNA (m^6^A-circRNA) microarray revealed the m^6^A-circRNA landscape in BC. M^6^A-modifid circATAD2 upregulated in BC samples and was closely correlated to poor prognosis. Functionally, circATAD2 promoted the immune evasion of BC cells and reduced the CD8^+^ T cells’ killing effect. Mechanistically, MeRIP-seq unveiled the m^6^A modification in the 3’-UTR of PD-L1 mRNA, which was bound by circATAD2 and recognized by m^6^A reader IGF2BP3 to enhance PD-L1 mRNA stability and expression. In summary, these findings revealed the circATAD2/m^6^A/IGF2BP3/PD-L1 axis in BC immune surveillance, suggesting the potential that circATAD2 as a potential target for PD-L1-mediated BC.

**Supplementary Information:**

The online version contains supplementary material available at 10.1007/s00262-024-03705-6.

## Introduction

Breast cancer (BC) is the most common malignant tumor among women worldwide [1, 2]. The incidence rate of BC observably increased, ranking as the second leading cause of cancer-related death in women [3, 4]. Tumor immunotherapy is a common chemical method for BC, however, the therapeutic efficiency of immunotherapy is seriously limited due to immunotherapy target failure.

 Immune surveillance is the function of immune system to identifying, killing and removing mutant cells in vivo timely to prevent tumor occurrence [5]. Immune surveillance acts as one of the most basic functions of human immune system, and its low function will form tumors [6]. Immune escape is the process of escape behavior produced by pathogens in response to human immune system attacks. BC immune evasion could give rise to secondary recurrent or metastatic tumors by transient amplifying differentiated BC cells. The current theory raises new viewpoints that one of the main reasons of BC’s recurrence and metastasis is BC immune evasion to CD8^+^ T cells. Balaji Virassamy et al. [7] put forward a perspective that antitumor immunity and immune checkpoint blockade effectiveness could induce BC immune evasion attributing to significantly enhanced cytotoxic capacity. Therefore, a better understanding of BC immune surveillance and immune evasion might be imperative for progressing BC targeted treatment.

The chemical modifications of RNA are a variety of regulation on RNA function. Among series of RNA modifications, N^6^-methyladenosine (m^6^A) acts as the most abundant and conserved chemical modification in mammalian cells [8]. Dynamically, m^6^A was installed/uninstalled by ‘writers’ (e.g. METTL3, METTL14, WTAP, KIAA1429) and ‘erasers’ (FTO, ALKBH5). Meanwhile, m^6^A modification is recognized by ‘readers’ (e.g. IGF2BP1/2/3, YTHDC1/2, YTHDF1/2/3, HNRNPA2B1) [9, 10]. The modification of m^6^A plays an essential role on RNA processing, nuclear export, mRNA stability and translation.

To investigate the potential function of m^6^A-modfied circATAD2 on BC immune evasion and CD8^+^ T cells-mediated immune surveillance, our work performed functional assays and mechanical experiments to investigate and confirm the pathway. In present research, m^6^A-modifid circRNA (m^6^A-circRNA) microarray revealed the m^6^A-circRNA landscape in BC cells. M^6^A-modifid circATAD2 (hsa_circ_0085465, 333 bp, ATAD2 exon 21-20) functioned as an oncogene in the BC tumorigenesis and participated in the BC immune evasion and CD8^+^ T cells-mediated immune surveillance through m^6^A-modification manner. PD-L1, a key factor in maintaining immune evasion, was found to be positively targeted by circATAD2 via IGF2BP3-dependent pathway. In conclusion, our research focused on investigating whether circATAD2-modulated BC immune evasion and CD8^+^ T cells-mediated immune surveillance, which are pivotal for immunotherapy.

## Materials and methods

### Clinical tissue subjects and specimens

A total of 45 clinical cases of breast cancer specimens were recruited during surgery at ShanXi Provincial People’s Hospital. The tumor tissue was graded or classified by two independent experienced pathologists. All patients’ participators had provided the informed consent, and all the procedure was approved by the Ethics Committee of ShanXi Provincial People’s Hospital.

### Cell lines and culture

All these cell lines, including BC cells (MDA-MB-231, MCF-7) and normal human breast epithelial cell (MCF-10A), were acquired by the American Type Culture Collection. All cells were cultured in Dulbecco’s modified Eagle’s Medium (DMEM, Gibco, Carlsbad, CA, USA) and supplemented with 10% FBS (fetal bovine serum, Gibco, Carlsbad, CA, USA) in atmosphere containing 5% CO_2_ at 37 °C.

### Transfection

To knockdown the expression of circATAD2 in BC cells, short hairpin RNA (shRNA) targeting circATAD2 with pLKO.1 constructs packing and pCMV delta R8.2 plasmids and pCMV-VSV-G were co-transfected into HEK293T cells. 72 h post-transfection, lentiviral particles were collected and then infected BC cells with polybrene (6 mg/mL). After 48 h infection, puromycin (4 mg/mL, Sigma-Aldrich, Cat no. 58-58-2) was added to select the infected cells. To upregulate the expression of circATAD2, human circATAD2 cDNA was cloned into pCDH puro lentiviral vector (CD510B-1, System Biosciences). Transfection of plasmids was performed using Lipofectamine 2000 (Invitrogen, catalog no. 11668027) according to the manufacturer’s instructions.

### RNA extraction and quantitative real‑time PCR assays

Total RNA from BC cell lines was extracted using TRIzol reagent (Invitrogen, CA, USA). cDNA was synthesized using SuperScript First-Stand Synthesis system (Invitrogen, USA), and real-time quantitative PCR (RT-qPCR) analysis was performed using the SYBR Green methods on ABI PRISM 7900 thermocycler according to the manufacturers’ instructions. The relative expression of mRNA was calculated using 2^–ΔΔ Ct^ method. The specific primers are listed in the Supplementary Table S1.

### Western blot assay

Western blot assay was performed based on previously described [11]. Briefly, each protein lysate (30 μg) was resolved using 10% sodium dodecyl sulfate-polyacrylamide gel electrophoresis (SDS-PAGE, Sigma-Aldrich, St Louis, MO, USA), and then transferred to PVDF membrane. The membrane was incubated with primary antibodies overnight at 4 °C. The blots were incubated with secondary antibodies and detected using the ECL chemiluminescent system. Lastly, the blots’ quantitative analysis for band intensity was carried out by ImageJ software. The gray value ratios of protein bands and internal control bands were regarded as the relative level. The experiments were repeated at last three times for mean value.

### Actinomycin D treatment and RNA stability assay

MCF-7 or MDA-MB-231 cells were treated with 1 μg/ml actinomycin D (Abcam) at indicated times [0, 3, and 6 h]. Then, total RNA of the BC cells was extracted. To identify circular characteristics of circATAD2 stability, the total RNA (2 μg) was incubated with or without RNase R (6 U, 3 U/μg) (Epicentre, SanDiego, CA, USA) at 37 °C for 30 min. The relative expression level of circATAD2, ATAD2 were determined using qRT-PCR.

### CD8^+^ T cells isolation

Human CD8^+^T cells were obtained from healthy donor peripheral blood mononuclear cells (PBMCs), which was purified by a CD8^+^ Cell Positive Selection Kit (ImunoSep, Precision) according to the manual’s protocol. For the activation of CD8^+^ T cells, cells were resuspended in RPMI 1640 medium and incubated with CD3/CD28 Dynabeads (2 µl/well) and IL-2 (10 ng/mL) for 48 h. The activated CD8^+^ T cells were used for the following co-culture assays (effector:targetor cells ratio was 5:1).

### ELISA assays, surface PD-L1 and cytotoxicity analysis

The quantified levels of IFN-γ, TNF-α, Granzyme B secreted by co-cultured activated CD8^+^ T cells were detected using ELISA kits, including Human TNF-α ELISA Kit, IFN-γ ELISA Kit (MultiSciences, China) and Human Granzyme B ELISA Kit (Beyotime Institute of Biotechnology) according to the manufacturer’s protocol. The supernatants were collected and detected by 450 nm Optical densities using microplate reader (Bio-Tek). For the surface PD-L1, ~ 80% confluency BC cells were stained by FAM-labeled PD-L1 antibody (BioLegend, USA). After washing by PBS three times, FAM-positive cells were detected by flow cytometry. The release of lactate dehydrogenase (LDH) in the supernatants was detected on cell lysis using the Cytotoxicity Detection Kit PLUS (Sigma-Aldrich) according to the manufacturer’s protocol.

### M^6^A quantification

The global m^6^A levels in mRNA were colorimetrically quantified by EpiQuik m^6^A RNA Methylation Quantification Kit (EpiGentek, cat. P-9005) according to manufacturer’s instruction.

#### RNA immunoprecipitation (RIP)

The RIP assay for molecular interaction was performed using Magna RIP Kit (Millipore, USA) according to the manufactures’ guidelines. In brief, 5 × 10^6^ BC cells were harvested and lysed in RIP buffer (20 mM Tris–HCl pH 7.5, 140 mM NaCl, 0.05% TritonX-100). After centrifugation in supernatant at 4 °C, antibody (5 μg, specific antibodies or negative control IgG) was pre-bound to Protein A/G magnetic beads and then incubated with 100 μl cell lysates over night with rotation. RNA was eluted from the beads by elution buffer and the eluted RNA was precipitated. Finally, the enrichment of mRNA fragments was determined by real-time PCR.

#### methylated RNA immunoprecipitation PCR (MeRIP-PCR)

To quantify the m^6^A-modified level of PD-L1 mRNA, MeRIP-PCR was performed as previously described[12]. Total RNA was isolated from BC cells by Trizol, and the extracted RNA was chemically fragmented into ~ 100 nt. RNA fragments were incubated with m^6^A antibody (Abcam, cat. ab151230) for immunoprecipitation according to the manufacture’s instruction. After removing the supernatant, cells were treated by 100 μL RIP lysis buffer and incubated with the lysate on ice. m^6^A antibody (5 μg) was added to magnetic beads and then rotated at room temperature for 30 min. Then, beads were washed with RIP wash buffer and resuspended in RIP immunoprecipitation buffer following by centrifugation at 14,000 rpm for 10 min. The enrichment of m^6^A-containing PD-L1 mRNA was validated by quantitative RT-PCR.

#### methylated RNA immunoprecipitation sequencing (MeRIP-Seq)

m^6^A-IP and library were prepared according to previously protocol [13]. Briefly, poly-A purified RNAs were fragmented and then incubated by anti-m^6^A primary antibody (Abcam, cat. ab151230) for 2 h. The mixture was immunoprecipitated via being incubated with Protein A beads (Thermo Fisher) and following buffer washing for 3 times to retain captured RNA. After elution, the RNA was purified by RNA Clean and Concentrator kit (Zymo, Darmstadt, Germany). Sequencing was performed on Illumina HiSeq 2000 according to the protocol’s instructions.

#### Luciferase reporter assay

The dual-luciferase reporter gene vectors containing PD-L1 mRNA 3’-UTR wild-type sequence and mutants were formulated, respectively (Luc-PD-L1-3’UTR-Mut, Luc-PD-L1-3’UTR-MUT). Two reporter plasmids, as well as sh-circATAD2/oe-circATAD2, were co-transfected into 293 T cells. After transfection, the cells were lysed and centrifuged at 12,000 rpm for 1 min. After the supernatant collection, the luciferase activity was detected by Dual-Luciferase® Reporter Assay System (Promega). Furthermore, firefly luciferase working solution was added to detect firefly luminescence (firefly luminescence) to identify Renilla luciferase (renilla luciferase).

#### mRNA stability analysis

Cells were exposed to Act D at a concentration of 8 μg/ml for 0 h, 3 h, 6 h. At different time points, the cells were collected and RNA was extracted using Trizol reagent (Invitrogen, Grand Island, NY, USA). After RNA extraction, the PD-L1 mRNA level was identified using RT-qPCR as described earlier.

#### In vivo mouse experiments

C57BL/6 mice were purchased (Vitalriver, Beijing, China) were randomly divided into two groups. Mice were subcutaneously injected in the right flank with BC cells (MCF-7, 2 × 10^6^ cells in 100 μl). All handling procedures for animal care were performed in accordance with the National Institutes of Health guide for Laboratory animals using and care. The animal assay had approved by the Ethics Committee of ShanXi Provincial People’s Hospital.

#### Statistical analysis

All experiments were performed in triplicate and data were presented as mean value ± standard deviation (SD) by SPSS statistical software. The data was statistically analyzed by one-way analysis of variance (ANOVA) or Student’s t-test. p < 0.05 was considered statistically significant. *p < 0.05, **p < 0.01.

## Results

### m^6^A-circRNA microarray revealed the m^6^A-modified circATAD2 in BC

**Fig. 1:**
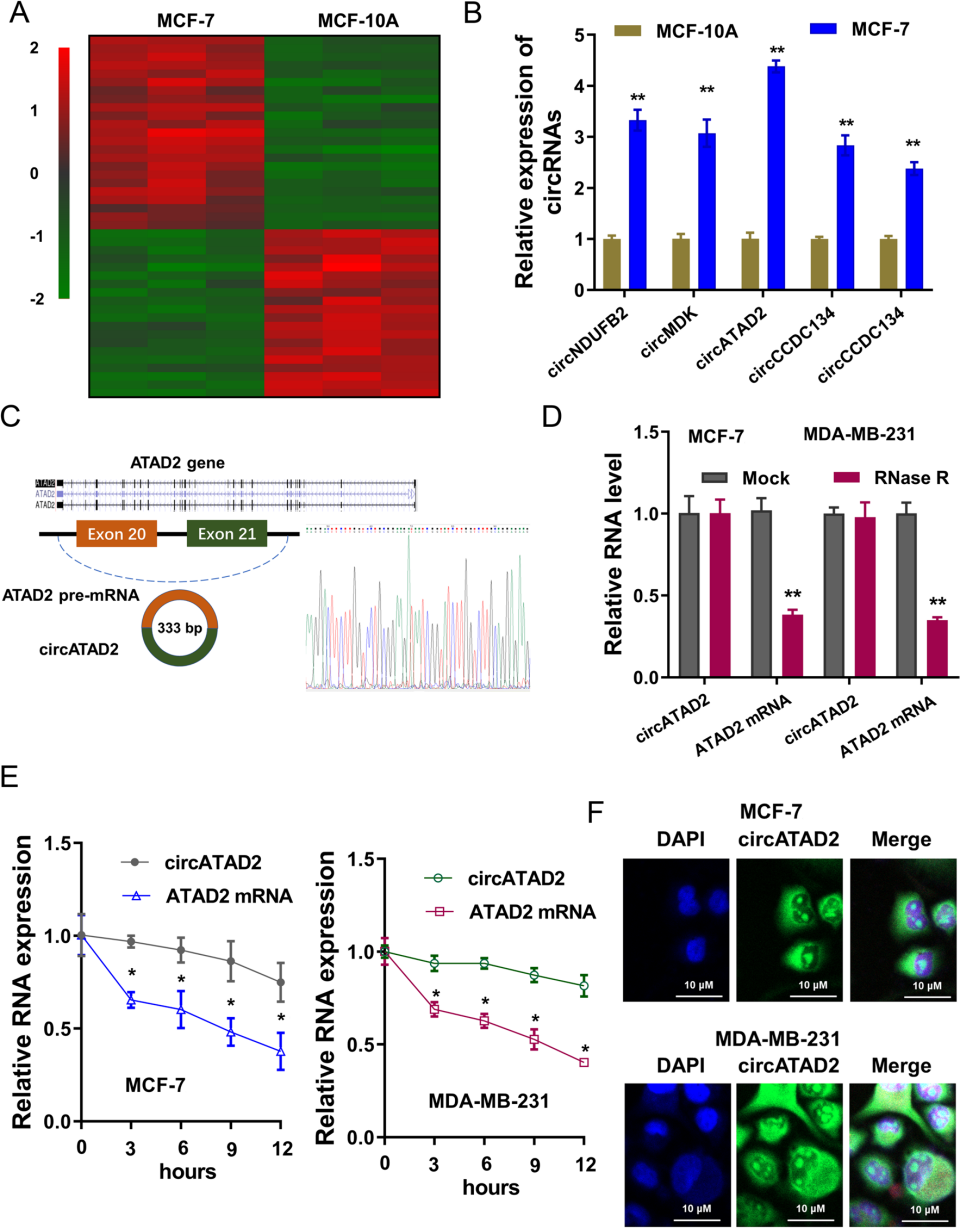
m^6^A-circRNA microarray revealed the m^6^A-modified circATAD2 in BC. **A** m^6^A-circRNA microarray assay was performed in BC cells (MCF-7) or normal cells (MCF-10A). Heatmap revealed the potential profile of m^6^A-modified circRNA landscape, including upregulated and downregulated m^6^A-circRNA. **B** Several possible circRNAs from the candidate m^6^A-circRNA, were detected for the relative expression. **C** Schematic diagram revealed the biogenesis of circATAD2 (hsa_circ_0085465, 333 bp) from exon 21- exon 20 of ATAD2 gene. Sanger sequencing revealed the co-junction site of circATAD2. **D** RNase R treatment assay was performed and the level of circular transcripts circATAD2 and linear ATAD2 mRNA using RT-PCR. **E** Actinomycin D assay was performed and the level of circular transcripts circATAD2 and linear ATAD2 mRNA using RT-PCR. **F** RNA cellular location analysis by RNA-FISH indicated the subcellular location of circATAD2 in BC cells (MCF-7, MDA-MB-231). **p < 0.01; *p < 0.05

To discovery the potential profile of m^6^A-modified circRNA landscape, the m^6^A-circRNA microarray assay was performed in BC cells (MCF-7) and normal cells (MCF-10A). The m^6^A-circRNA landscape was displayed as following, including upregulated and downregulated m^6^A-circRNA (Fig. 1A). From the candidate m^6^A-circRNA, several possible circRNAs were selected and detected for the relative expression, showing the higher expression of m^6^A-modified circATAD2 (Fig. 1B). M^6^A-modifid circATAD2 (hsa_circ_0085465, 333 bp) was derived from exon 21- exon 20 of ATAD2 gene (Fig. 1C). To identify circular characteristics of circATAD2 stability, RNase R treatment assay showed that circATAD2 was more stable than the linear ATAD2 mRNA (Fig. 1D). Actinomycin D assay revealed that circATAD2 was also more stable than the linear ATAD2 mRNA (Fig. 1E). RNA cellular location analysis by RNA-FISH indicated that circATAD2 located in the cytoplasm of BC cells (MCF-7, MDA-MB-231) (Fig. 1F). Thus, m^6^A-circRNA microarray revealed the m^6^A-modified circATAD2 in BC.

### circATAD2 upregulated in BC and acted as an oncogenic factor

**Fig. 2:**
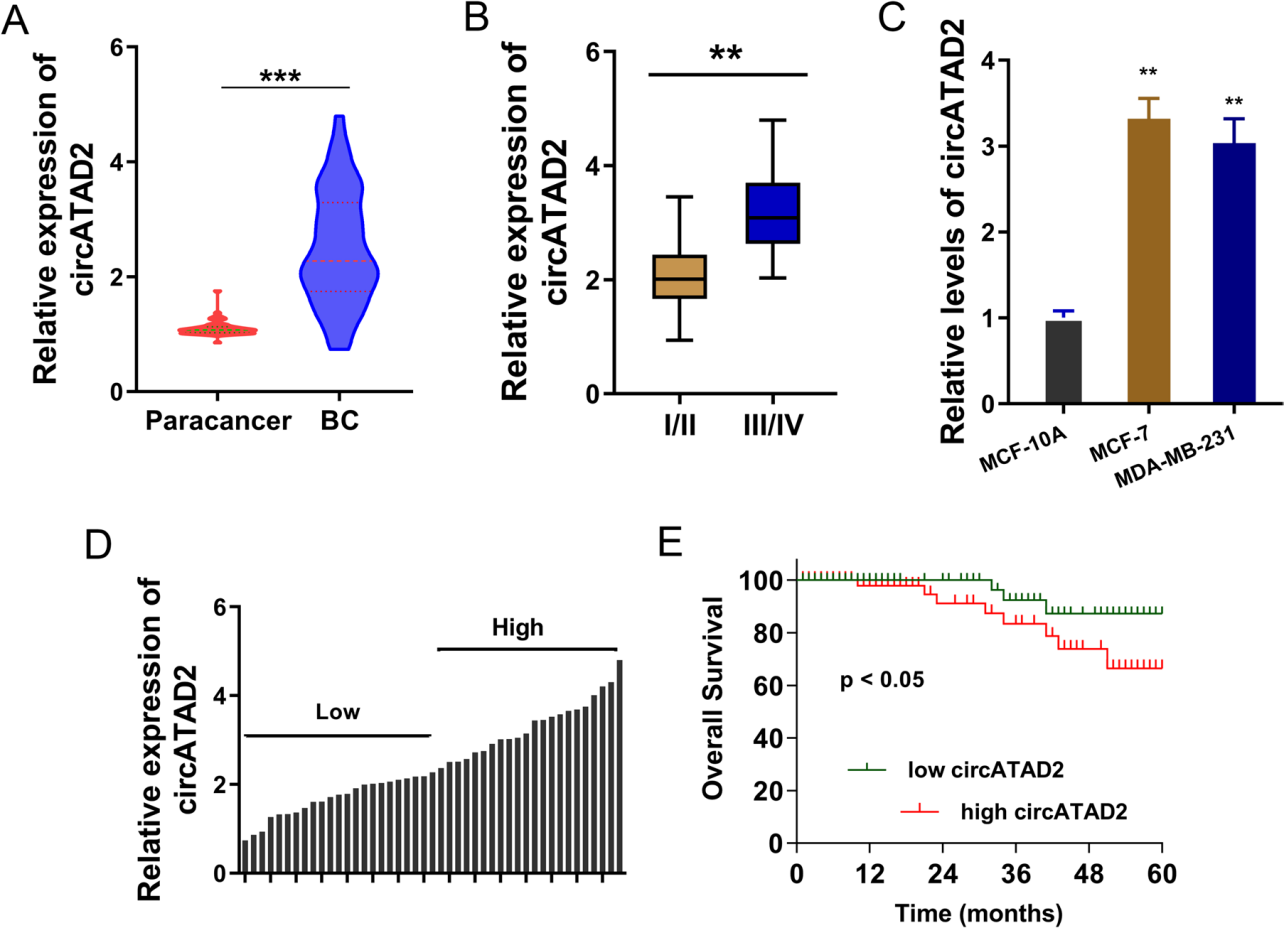
circATAD2 upregulated in BC and acted as an oncogenic factor. **A** The circATAD2 levels were detected by RT-PCR in BC samples and control normal samples. **B** The level of circATAD2 in BC advanced stage (III/IV phase) and the initial stage (I/II phase). **C** CircATAD2 expression levels were detected using RT-PCR in the BC cells (MCF-7, MDA-MB-231). **D** The BC patients were divided into high group and low group based on the median value. **E** The survival rate of BC patients with high-circATAD2 exerting lower overall survival rate and low-circATAD2 exerting higher overall survival rate. **p < 0.01; ***p < 0.001

Given that m^6^A methylated level always upregulated in multiple tumors, including BC. Here, we detected m^6^A-modified circATAD2 to investigate its function and mechanism on BC. In BC samples, circATAD2 levels significantly upregulated as comparing to normal samples (Fig. 2A). Moreover, the level of circATAD2 upregulated in BC advanced stage (III/IV phase) than the initial stage (I/II phase) (Fig. 2B; Table 1). CircATAD2 expression levels were upregulated in the BC cells (MCF-7, MDA-MB-231) (Fig. 2C). The BC patients were divided into high group and low group based on the median value (Fig. 2D). For the clinical outcome, the survival rate showed that the patients with high-circATAD2 exerted lower overall survival rate (Fig. 2E). Taken together, these data suggested that circATAD2 upregulated in BC and acted as an oncogenic factor.

**Table 1:** Interaction of circATAD2 with breast cancer patients’ clinicopathological features

	N	circATAD2		*P* value
		Low = 22	High = 23	
Age				
< 50 years	19	8	11	0.4364
≥ 50 years	26	14	12	
Distant metastases				
Yes	25	11	14	0.4632
No	20	11	9	
TNM stage				
I–II	17	12	5	0.0233*
III–IV	28	10	18	
Lymph node metastasis				
Yes	27	10	17	0.0514
No	18	12	6	
Tumor size				
< 2 cm	16	11	5	0.0477*
≥ 2 cm	29	11	18	
HR status				
Positive	22	12	10	0.4578*
Negative	23	10	13	
HER-2 status				
Positive	18	9	9	0.9031
Negative	27	13	14	

### circATAD2 impaired CD8^+^ T cells-mediated BC immune surveillance

**Fig. 3:**
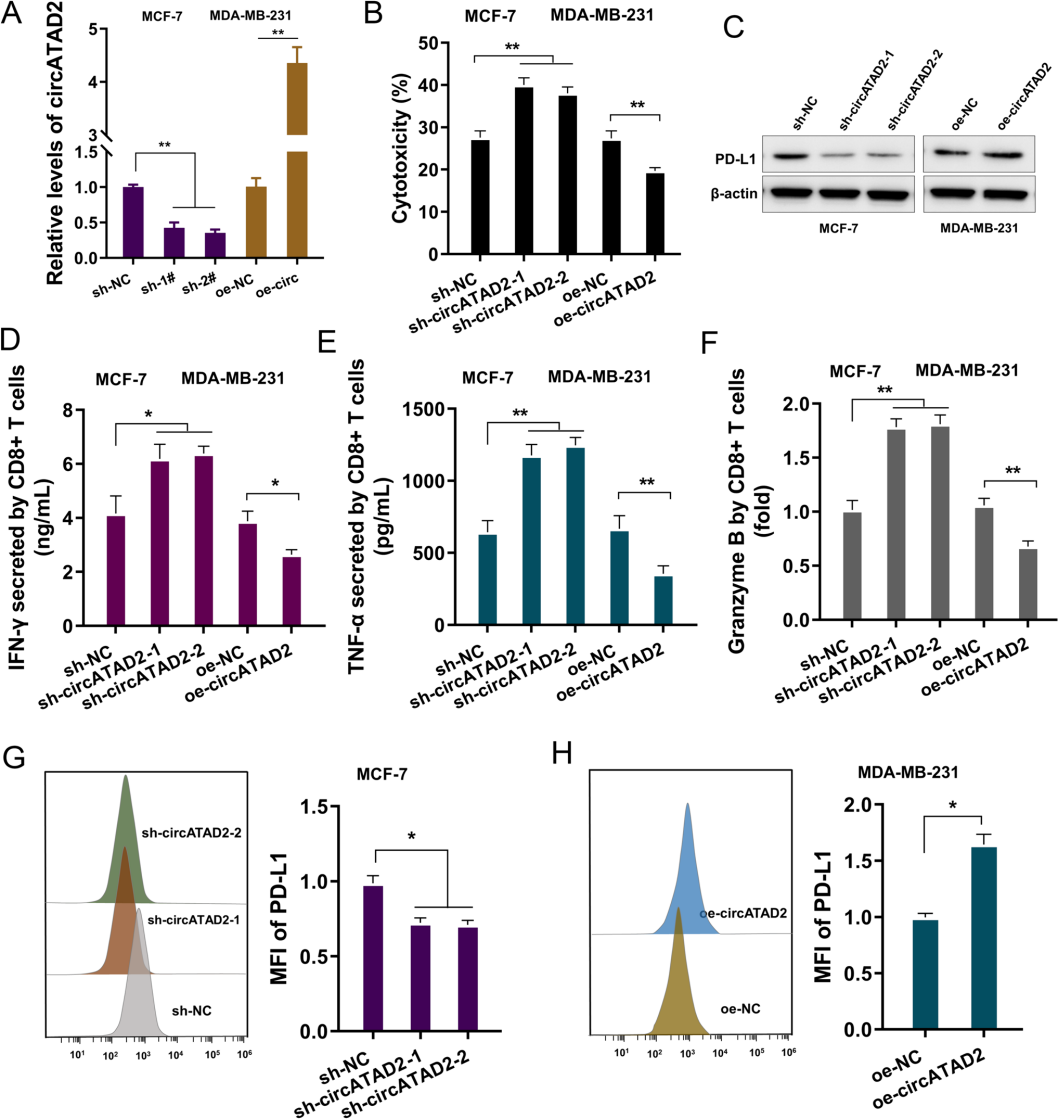
circATAD2 impaired CD8^+^ T-mediated BC immune surveillance. **A** The knockdown/silencing and overexpression of circATAD2 were constructed and detected using RT-PCR. **B** The CD8^+^ T cell-mediated cytotoxicity on BC cells was determined by lactate dehydrogenase (LDH) release assay in BC cells (MCF-7, MDA-MB-231). **C** Western blot assay was performed to detect the PD-L1 protein expression. **D**–**F** ELISA for IFN-γ, TNF-α and granzyme B levels were detected according to the manufacturer’s instructions. **G**, **H** Flow cytometry assay was performed for the PD‑L1 surface analysis of BC cells was performed with circATAD2 silencing and circATAD2 overexpression in coculture with activated CD8^+^ T cells. **p < 0.01; *p < 0.05

To investigate the function of circATAD2 on BC immune evasion, co-culture assays were performed using CD8 + T cells with BC cells with circATAD2 silencing or overexpression. Firstly, the knockdown/silencing and overexpression of circATAD2 were constructed, and the efficient was detected (Fig. 3A). Cytotoxicity assay detecting LDH released from the co-cultured culture supernatant showed that circATAD2 silencing accelerated the cytotoxicity, and circATAD2 overexpression repressed the cytotoxicity (Fig. 3B). PD-L1 protein was detected and results illustrated that circATAD2 silencing inhibited the PD-L1 expression, while circATAD2 overexpression accelerated the PD-L1 expression (Fig. 3C). In activated CD8^+^ T cells co-cultured with BC cells, IFN-γ, TNF-α and granzyme B levels were detected and results showed that circATAD2 silencing promoted the IFN-γ, TNF-α and granzyme B levels, while circATAD2 overexpression repressed the IFN-γ, TNF-α and granzyme B levels (Fig. 3D–F). Surface PD-L1 level was detected and results indicated that circATAD2 silencing reduced the surface PD-L1 level, while circATAD2 overexpression facilitated surface PD-L1 level (Fig. 3G, H). Thus, these data indicated that circATAD2 repressed the antitumor immunity of CD8^+^ T cells toward BC cells, thereby impairing CD8^+^ T cells-mediated BC immune surveillance.

### PD-L1 was a m.^6^A-modified target of circATAD2

**Fig. 4:**
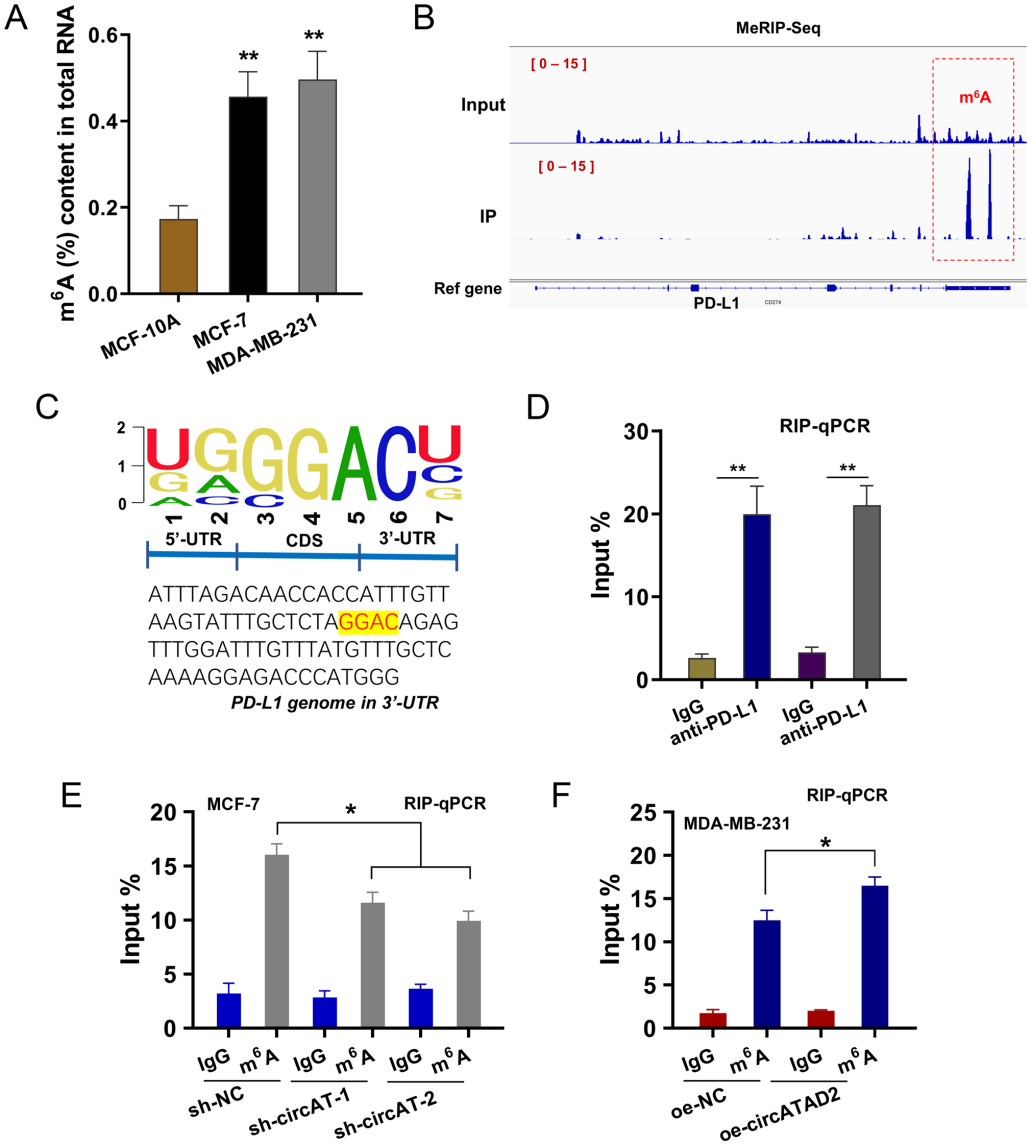
PD-L1 was a m^6^A-modified target of circATAD2. **A** The m^6^A quantitative analysis showed the m^6^A-modified enrichment in BC cells and normal cells. **B** The m^6^A-modified peaks were identified on the 3’-UTR of PD-L1 genomic based on MeRIP-Seq. **C** Schematic diagram demonstrated the location in the PD-L1 gene. **D** RIP-PCR assay illustrated the circATAD2 enriched by anti-PD-L1 antibody in BC cells (MCF-7, MDA-MB-231). **E**, **F** RIP-PCR demonstrated the PD-L1 mRNA enriched by anti-m^6^A antibody in BC cells (MCF-7, MDA-MB-231). **p < 0.01; *p < 0.05

The m^6^A-modified enrichment was found to be increased in BC cells (Fig. 4A). In the MeRIP-Seq, the m^6^A-modified peaks were identified on the 3’-UTR of PD-L1 genomic (Fig. 4B). The m^6^A-modified motif on the PD-L1 mRNA 3’-UTR was GGAC (Fig. 4C). RIP-PCR assay illustrated that the PD-L1 mRNA was enriched by anti-circATAD2 antibody (Fig. 4D). The m^6^A-modified enrichment analysis revealed that circATAD2 silencing reduced the m^6^A content, and circATAD2 overexpression increased the m^6^A content (Fig. 4E), and circATAD2 overexpression promoted the m^6^A-modified PD-L1 mRNA level (Fig. 4F). Taken together, these data suggested that PD-L1 was a m^6^A-modified target of circATAD2.

### IGF2BP3 enhanced the stability of PD-L1 mRNA

**Fig. 5:**
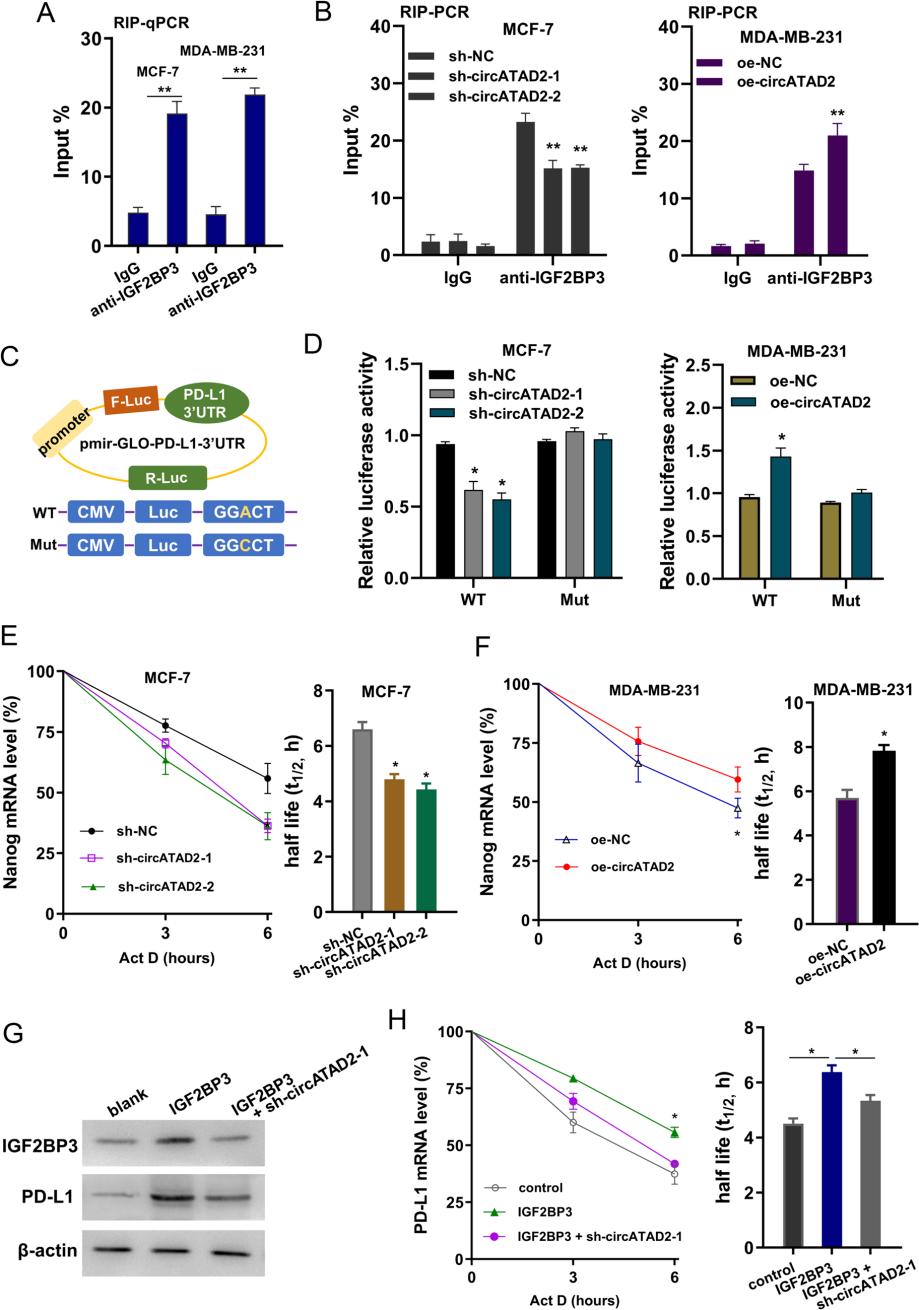
IGF2BP3 enhanced the stability of PD-L1 mRNA. **A** RIP-PCR was performed using anti-IGF2BP3 antibody in BC cells (MCF-7, MDA-MB-231) with knockdown/silencing and overexpression of circATAD2. **B** RIP-PCR illustrated the PD-L1 mRNA precipitated by anti-IGF2BP3 antibody in BC cells (MCF-7, MDA-MB-231) with knockdown/silencing and overexpression of circATAD2. **C** Schematic diagram demonstrated the sequence of PD-L1 3’-UTR sequence for luciferase reporter assay (wild-type, WT or mutant, Mut). **D** Luciferase reporter assay was performed and the luciferase activity was computed by the ratio of Firefly and Renilla luciferase values. **E**, **F** RNA decay analysis showed the RNA half-life (t_1/2_) time of PD-L1 mRNA in BC cells (MCF-7, MDA-MB-231) treated by Act D. **G** Western blot analysis showed the protein level of IGF2BP3 and PD-L1 in MCF-7 cells. (H) RNA decay analysis showed the RNA half-life (t_1/2_) time of PD-L1 mRNA in MCF-7 cells. **p < 0.01; *p < 0.05

To explore how the m^6^A modification regulated the PD-L1 phenotype, we detected the potential m^6^A readers to find the targets. In our previous research, we noticed that IGF2BP3 could closely correlated to the PD-L1 mRNA in BC cells (Fig. 5A). Moreover, RIP-PCR illustrated that circATAD2 silencing reduced the PD-L1 mRNA enrichment precipitated by anti-IGF2BP3 antibody, while circATAD2 overexpression promoted the PD-L1 mRNA precipitated by anti-IGF2BP3 antibody (Fig. 5B). Luciferase reporter assay using PD-L1 mRNA plasmids (wild-type, WT or mutant, Mut of 3’-UTR sequence) illustrated that circATAD2 modulated PD-L1 expression mainly through the m^6^A site, and circATAD2 silencing reduced the activity of WT 3’-UTR luciferase reporter gene, and vice versa (Fig. 5C, D). RNA decay analysis found that circATAD2 silencing inhibited the RNA half-life (t_1/2_) time of PD-L1 mRNA, and circATAD2 overexpression upregulated the half-life (t_1/2_) time of PD-L1 mRNA (Figs. 5E, 7F). The IGF2BP3 upregulation transfection was constructed to test the role of circATAD2/IGF2BP3 on PD-L1 mRNA (Fig. 5G). Then, RNA decay analysis found that IGF2BP3 increased the half-life (t_1/2_) time of PD-L1 mRNA, and co-transfection (IGF2BP3 + sh-circATAD2) recovered the half-life (t_1/2_) time (Fig. 5H). Taken together, these data suggested that IGF2BP3 enhanced the stability of PD-L1 mRNA.

### circATAD2/m^6^A/IGF2BP3/PD-L1 axis impairs CD8^+^ T cells-mediated breast cancer immune surveillance

**Fig. 6:**
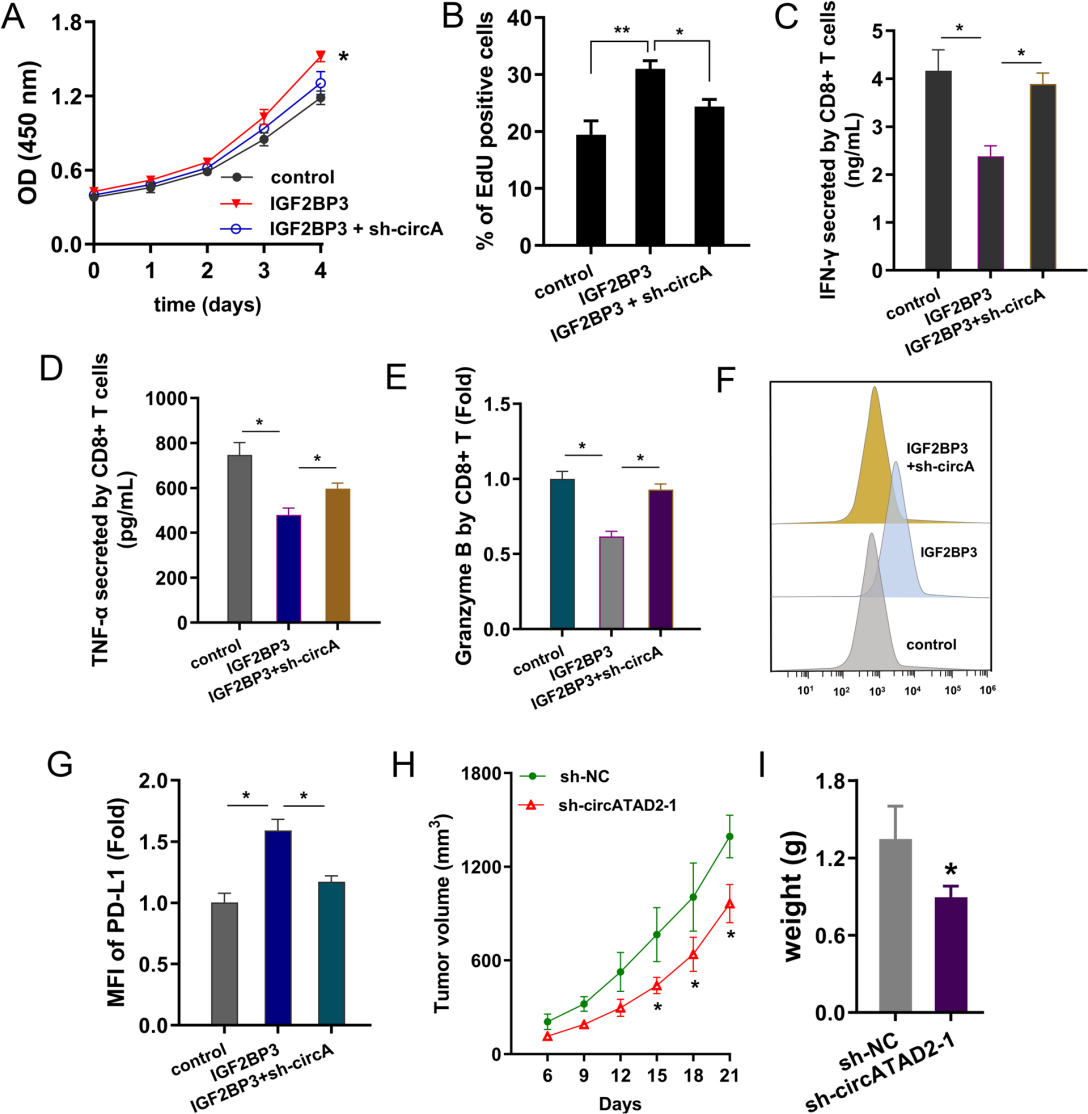
circATAD2/IGF2BP3/PD-L1 axis potentiates breast cancer immune evasion. **A** CCK-8 and **B** EdU assays were performed to detect the viability of MCF-7 with IGF2BP3 overexpression (IGF2BP3) and co-transfection of circATAD2 silencing (IGF2BP3 + sh-circATAD2-1). **C–E** ELISA for IFN-γ, TNF-α and granzyme B levels were performed in MCF-7 cells. **F**, **G** The flow cytometry assay for PD‑L1 surface of BC cells was performed in MCF-7 cells. MFI: mean fluorescence intensity. **H**, **I** The in vivo mice assay reflected the tumor volume and weight. **p < 0.01; *p < 0.05

**Fig. 7:**
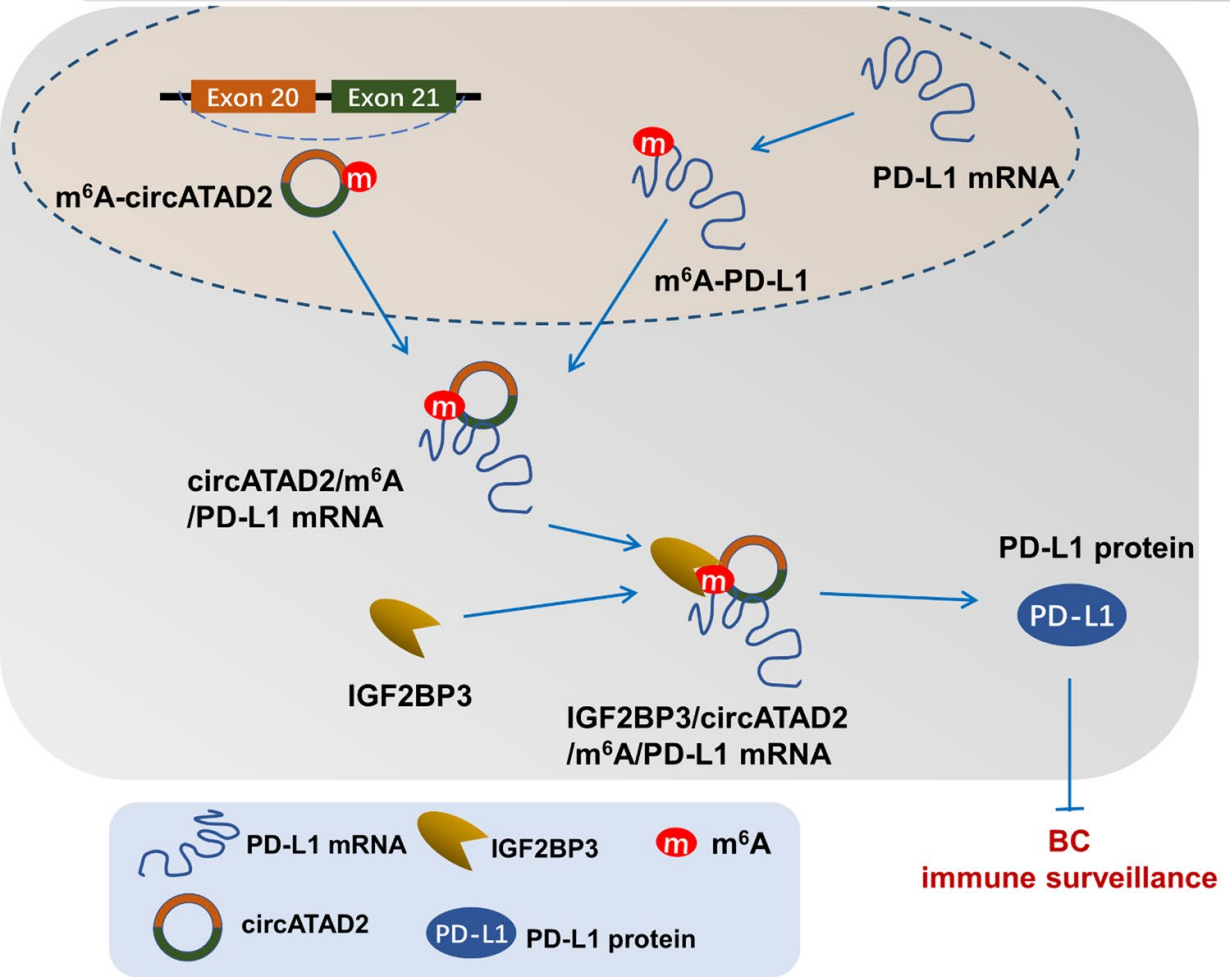
circATAD2 contributes to CD8^+^ T cells-mediated immune surveillance via m^6^A/IGF2BP3/PD-L1 axis, providing a potential target for BC

To confirm the role of circATAD2/IGF2BP3/PD-L1 axis on BC immune evasion, rescue assays were performed. Firstly, viability analysis by CCK-8 and EdU assays showed the IGF2BP3 upregulated the viability and circATAD2 silencing co-transfection recovered it (Fig. 6A, B). Besides, IGF2BP3 downregulated the IFN-γ, TNF-α and granzyme B levels in MCF-7 cells, while circATAD2 silencing co-transfection recovered the IFN-γ, TNF-α and granzyme B levels (Fig. 6C–E). Moreover, IGF2BP3 upregulated the surface PD-L1 level on BC cells, while circATAD2 silencing co-transfection recovered the surface PD-L1 level (Fig. 6F). Besides, the in vivo assays suggested that circATAD2 silencing repressed the tumor growth (Fig. 6G). Besides, circATAD2 silencing repressed the tumor growth in vivo (Fig. 6H, I). Overall, these data suggested that circATAD2 contributes to CD8^+^ T cells-mediated immune surveillance via m^6^A/IGF2BP3/PD-L1 axis (Fig. 7).

## Discussion

Recently, circRNA and m^6^A regulators have been identified to play crucial roles during BC tumorigenesis and cancer progression [14–18]. However, their functional role and underlying molecular mechanism in BC immune evasion and immune surveillance remain unclear [19, 20]. Based on our initial investigation, a novel m^6^A-modified circATAD2 acts as a potential oncogenic role in BC.

Here, we carried out a series of functional assays and results revealed that circATAD2 was elevated in BC cells and tissue samples, suggesting a potential oncogenic role on BC. Here, our results showed that circATAD2 regulated CD8^+^ T cells-mediated immune surveillance and BC immune evasion. Accumulating evidences have showed that targeting BC immune evasion is of great benefit to improve the clinical therapeutic effect for BC [11, 21]. BC immune evasion is generally defined as a group of xenogeneic cells that could get rid of the immune system killing and give rise to a tumor recrudesce.

Here, the functions of circATAD2 are straightforward as oncogene. We evidenced that in vivo and in vitro experiments to systemically delineate the biological functions of circATAD2 and its potential mechanism. In present research, circATAD2 has been identified as an essential oncogene in BC’s CD8^+^ T cells-mediated immune surveillance. A large number of previous studies also confirm that circRNA could regulate the BC immunoregulation. For instance, circular RNA hsa_circ_0001598 promotes BC trastuzumab-resistance, cell growth, PD-L1 expression and escapes CD8^+^ T cell killing via PD-L1-mediated immune escape [22]. Taken together, our findings suggest that the upregulation of novel circATAD2 contributed to impair CD8^+^ T cells-mediated immune surveillance and antitumor immunity of BC. Currently, tumor-specific cytotoxic T lymphocytes (CTLs) show better therapeutic effect on BC. In present research, it will be better to utilize tumor-specific CTLs in the co-culture system.

BC immune evasion plays essential and critical roles in BC formation, relapse, drug resistance and metastasis. PD-L1 plays a critical role in tumor immune evasion and maintenance [23, 24]. The major functions of PD-L1 depends on its transcriptional and post-transcriptional modifications in tumor cells. The findings discovered that circATAD2 was elevated in BC and increased PD-L1 mRNA stability and expression to maintain immune evasion. There are numerous literatures reporting that PD-L1 acts as a remarkable activator for BC. One of main characteristics for PD-L1 is that PD-L1 mediates BC immune evasion. In BC, PD-L1 could stimulate the immune evasion. For example, Takahiro Maeda et al. [25] reported that targeting MUC1-C associated with PD-L1 suppression could increase tumor-infiltrating CD8^+^ T cells quantity and tumor cell killing ability, which inversely correlates to CD8, CD69, and GZMB markers. Overall, the upregulation of novel circATAD2 contributed to impair CD8^+^ T cells-mediated antitumor immunity and immune surveillance of BC through enhancing RNA stability of PD-L1.

In summary, our findings suggest that chemotherapy elicited circATAD2 upregulated in the BC and was closely related to immunosuppressive microenvironment and CD8^+^ T cells-mediated immune surveillance. Furthermore, we reported a m^6^A-dependent mechanism whereby immune surveillance was inactivated in BC tumorigenesis. These data revealed the role of circATAD2 in CD8^+^ T cells-mediated antitumor immunity and immune surveillance, suggesting the potential of circATAD2 for BC immunotherapy.

### Supplementary Information

Below is the link to the electronic supplementary material.

Supplementary file1 (DOCX 17 kb)

## Data Availability

Not applicable.
