# Supplementary material for: circATAD2 mitigates CD8+ T cells antitumor immune surveillance in breast cancer via IGF2BP3/m6A/PD-L1 manner
Source: Cancer Immunol Immunother. 2024 May 15;73(7):130. doi: 10.1007/s00262-024-03705-6 (PMC11096152; doi:10.1007/s00262-024-03705-6)
Supplement: Supplementary file 1 — Supplementary file1 (DOCX 17 kb) [file 262_2024_3705_MOESM1_ESM.docx]

**supplement Table S1**. Primers sequences for qRT-PCR and sequences of shRNA.

|  | Sequences |
| --- | --- |
| circATAD2 | F, 5’- TGCTATTGACAAGCGATTCCG-3’  R, 5’-GTCCGTTCTTCTTTATCCGGT-3’ |
| PD-L1 | F, 5’- GCTGCACTAATTGTCTATTGGGA-3’  R, 5’- AATTCGCTTGTAGTCGGCACC-3’ |
| sh-circATAD2-1 | 5’- GACCCTGATGAGGTGCAAGAA-3’ |
| sh-circATAD2-2 | 5’- GATGAGGTGCAAGAATTGTTT-3’ |
| IGF2BP3 | F 5’- ACGAAATATCCCGCCTCATTTAC-3’  R 5’- GCAGTTTCCGAGTCAGTGTTCA-3’ |
| GAPDH | F 5’- GGAGCGAGATCCCTCCAAAAT-3’  R 5’- GGCTGTTGTCATACTTCTCATGG-3’ |
| Actin | F, 5’- CTCCATCCTGGCCTCGCTGT-3’  R, 5’- GCTGTCACCTTCACCGTTCC-3’ |
